# The impact of different exercise protocols on rat soleus muscle reinnervation and recovery following peripheral nerve lesion and regeneration

**DOI:** 10.3389/fphys.2022.948985

**Published:** 2022-09-06

**Authors:** Michael Di Palma, Patrizia Ambrogini, Davide Lattanzi, Lorenza Brocca, Roberto Bottinelli, Riccardo Cuppini, Maria A. Pellegrino, Stefano Sartini

**Affiliations:** ^1^ Department of Experimental and Clinical Medicine, Università Politecnica Delle Marche School of Medicine, Ancona, Italy; ^2^ Department of Biomolecular Sciences, University of Urbino “Carlo Bo”, Urbino, Italy; ^3^ Department of Molecular Medicine, University of Pavia, Pavia, Italy; ^4^ National Neurological Institute C. Mondino Foundation, Pavia, Italy; ^5^ Interdepartmental Centre of Biology and Sport Medicine, University of Pavia, Pavia, Italy

**Keywords:** peripheral nerve lesion, exercise, terminal axon sprouting, muscle reinnervation, autophagy, motor recovery

## Abstract

**Background:** Incomplete functional recovery following traumatic peripheral nerve injury is common, mainly because not all axons successfully regenerate and reinnervate target muscles. Exercise can improve functional outcomes increasing the terminal sprouting during the muscle reinnervation. However, exercise is not a panacea per se. Indeed, the type of exercise adopted dramatically impacts the outcomes of rehabilitation therapy. To gain insight into the therapeutic effects of different exercise regimens on reinnervation following traumatic nerve lesion, we evaluated the impact of different clinically transferable exercise protocols (EPs) on metabolic and functional muscle recovery following nerve crush.

**Methods:** The reinnervation of soleus muscle in adult nerve-crushed rats was studied following 6 days of different patterns (continuous or intermittent) and intensities (slow, mid, and fast) of treadmill running EPs. The effects of EPs on muscle fiber multiple innervation, contractile properties, metabolic adaptations, atrophy, and autophagy were assessed using functional and biochemical approaches.

**Results:** Results showed that an intermittent mid-intensity treadmill EP improves soleus muscle reinnervation, whereas a slow continuous running EP worsens the functional outcome. However, the mid-intensity intermittent EP neither enhanced the critical mediators of exercise-induced metabolic adaptations, namely, PGC-1α, nor improved muscle atrophy. Conversely, the autophagy-related marker LC3 increased exclusively in the mid-intensity intermittent EP group.

**Conclusion:** Our results demonstrated that an EP characterized by a mid-intensity intermittent activity enhances the functional muscle recovery upon a nerve crush, thus representing a promising clinically transferable exercise paradigm to improve recovery in humans following peripheral nerve injuries.

## Introduction

Despite the well-documented ability of motor neuron axons to regenerate and reinnervate peripheral targets, a complete muscle functional recovery following traumatic nerve lesion occurs in only 10% of patients (Scholz et al., 2009; Kamble et al., 2019). Considering that most injured people are between 20 and 40 years old, poor skeletal muscle recovery poses a significant public health concern. Failing of muscle motor-sensory recovery depends on different factors: 1) the number of regenerating axons able to reach the target based on the severity of the lesion; 2) the time required for nerve regeneration affecting the reinnervation of muscle far from the nerve lesion site; 3) the axon regeneration misdirection; 4) the surgical strategy to repair of nerve lesion (end-to-end repair, neural tube, nerve graft, nerve transfer, and so forth) (Asensio-Pinilla et al., 2009; Gordon et al., 2015; Kachramanoglou et al., 2017).

Skeletal muscle reinnervation occurs by the nerve-terminal sprouting of a limited number of regenerated axons (Rafuse and Gordon, 1996), resulting in an enlargement of motor unit size, which is insufficient to induce complete muscle reinnervation and functional motor recovery in rats (Sartini et al., 2013). Therefore, optimizing the axon sprouting process could represent a strategy to promote a wider multiple innervation of muscle cells prodromal to the 1:1 restoration of the nerve terminal/muscle fiber ratio of a larger muscle area, thus bolstering the functional recovery after a peripheral nerve injury. In line with this reasoning, our previous work (Sartini et al., 2013) showed that an intermittent exercise protocol carried out during muscle reinnervation positively affects muscular functional recovery by improving axonal plasticity in nerve-crushed rats (Sartini et al., 2013). The effect of this pattern of exercise on muscle reinnervation and functional recovery was, in part, accomplished by an increased muscle expression of BDNF and TrkB receptor involvement.

In this context, and in order to gain insight into the therapeutic effects of neuromuscular activity during and following the functional re-education of patients undergoing traumatic nerve lesion, it is essential to highlight which pattern and intensity of exercise can boost the metabolic and functional muscle recovery after a peripheral nerve injury.

Therefore, in the current work, using an ensemble of biochemical and functional approaches, we studied the effect of different patterns and intensities of exercise on adult rat soleus muscle re-innervation after nerve crush.

## Materials and methods

### Surgery and experimental groups

Experiments were carried out in accordance with the Italian law on animal experiments (D.lgs 26/2014; research project permitted with authorization N° 149/2018-PR by Italian Ministry of Health). Male Sprague-Dawley rats (Charles River Laboratory; *n* = 52) were kept on a 12/12 h day–night cycle, with food and water available *ad libitum*. Animals of 1.5 months of age were anesthetized using sodium thiopental (45 mg/kg b.w.) *via* i.p., and the soleus branch of the left tibial nerve was crushed 4 mm proximally from the muscle entry point, so that the first neuromuscular synaptic contacts are formed 3–4 days after muscle denervation (Favero et al., 2010).

**FIGURE 1:**
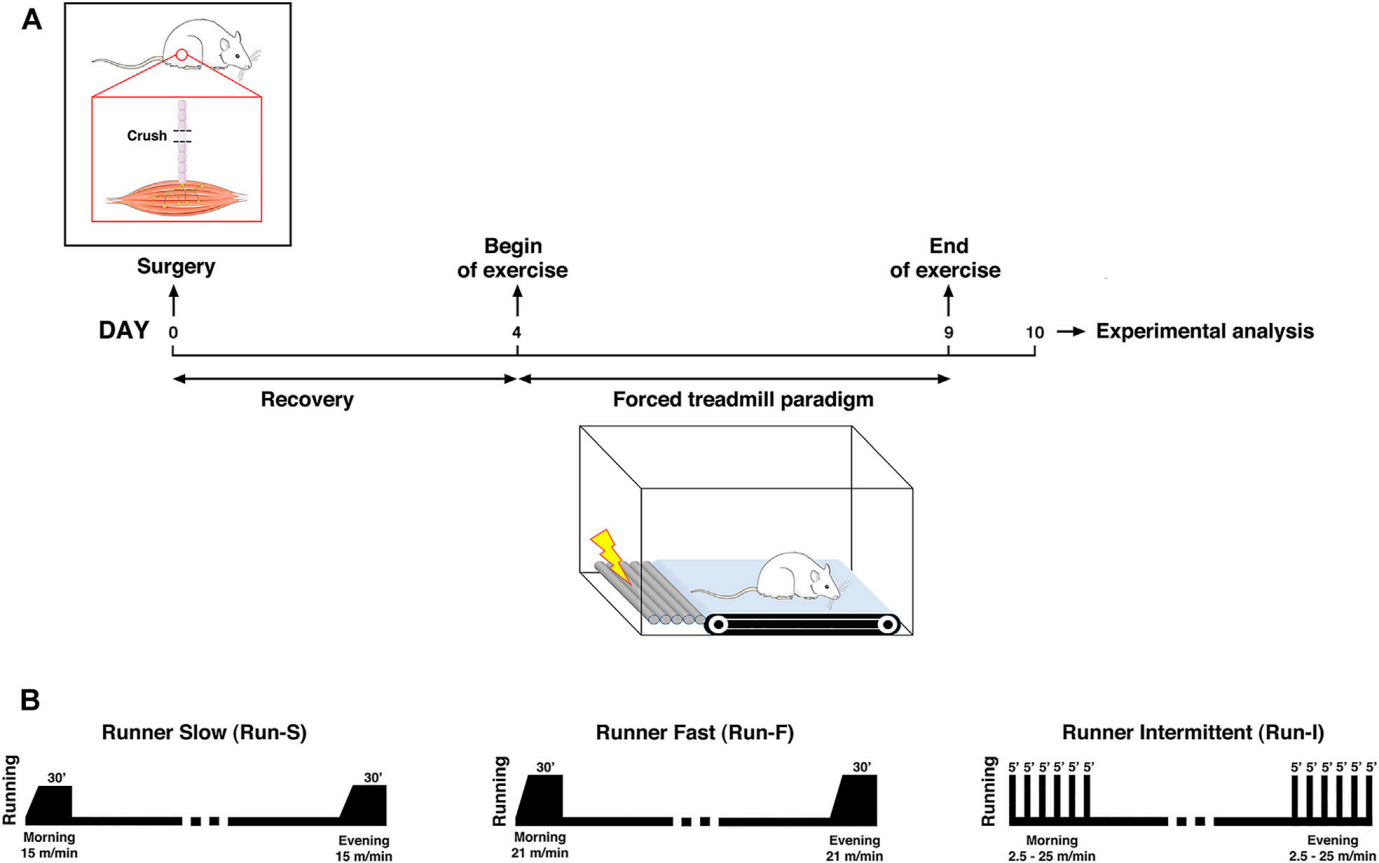
Schematic representation of the experimental design for assessing the exercise-induced effect on muscle re-innervation after nerve-crush **(A)** and exercise protocols used **(B)**.

Notably, the soleus muscle function is redundant with the gastrocnemius one. Thereby, its denervation does not compromise the animal’s locomotion activity. For this reason, it is a suitable denervation model to carry out motor activity programs following the injury. Therefore, 4 days after nerve crush, rats underwent exercise using a motor-driven treadmill twice a day for 6 days, after which animals were sacrificed for examination. In particular, this end point at 10 days from nerve crush was chosen according to our previous work showing that the most significant differences between exercised and control groups in the muscle reinnervation process occurred at this time point (Sartini et al., 2013). Animals were divided into the following experimental groups based on the exercise protocol used: 1) runner intermittent group (Run-I; *n* = 12), exercised for 30 min with an intermittent mid-intensity activity characterized by alternating 5-min periods of running and rest, with each a running period of 5 min consisting of 4 min at constant acceleration (0–27 m/min) and 1 min at the maximum speed of 27 m/min (Sartini et al., 2013); 2) runner fast group (Run-F; *n* = 10), trained for 30 min of continuous running at 21 m/min; and 3) runner slow group (Run-S; *n* = 10), exercised for 30 min of continuous running at 15 m/min. Exercise sessions were always carried out at the same hours (9 a.m. and 4 p.m.). Finally, sedentary controls (Sed; *n* = 15) were nerve-crushed rats kept in standard cages without exercise until examination, whereas undenervated controls (Und; *n* = 5) were rats without any surgery and kept in a standard cage (for a schematic representation of the experimental design and exercise protocols used; see Figure 1).

### *In vivo* tension recordings

Ten days after nerve crush, rats (*n* = 28) were anesthetized, as described above, and placed on a suitable support, with the hind legs positioned so that the soleus muscle was horizontally oriented. The left soleus muscle was exposed, and the distal tendon was cut and connected to an isometric tension transducer. The tibial nerve was isolated and cut upstream from the crushed point. Muscle contraction was elicited stimulating the nerve distal stump by a suction electrode (indirect stimulation) or muscle by a platinum-plate electrode (direct stimulation), previously adjusting the muscle length to obtain the maximum twitch tension. The nerve was stimulated at 100 Hz for 1 s to obtain a tetanic complete muscle response, and then the muscle was directly stimulated using the same protocol. Muscle contractions were monitored on an oscilloscope, stored on a computer hard-drive using an analogue-digital converter, and analyzed off-line by WinWCP software. Finally, a muscle innervation index was calculated as [strength obtained by nerve stimulation]/[strength obtained by muscle stimulation] ratio as previously described (Sartini et al., 2013).

### Electrophysiology

At the end of tension recording proceedings, rats were killed by an overdose of sodium thiopental, and soleus muscles were carefully removed together with a tract of its nerve supply and put into a recording chamber, containing Ringer’s solution bubbled with a mixture of O_2_ (95%) and CO_2_ (5%), in order to perform intracellular muscle recordings, as previously described (Sartini et al., 2013). Briefly, excitatory postsynaptic potentials (EPSPs) were intracellularly recorded following nerve stimulation with a glass microelectrode filled with 3 M KCl, using a DUO 773 amplifier (World Precision Instruments, United States) and WinWCP software (Strathclyde electrophysiology software V 4.1.3, John Dempster, University of Strathclyde, United Kingdom). Action potentials and contractions were prevented adding d-tubocurarine (10^–7^–10^–6^ g/ml) to the perfusion bath. Muscle cells were considered to be multiply innervated when two or more EPSPs were recorded with a different threshold or latency when the nerve stimulus strength was increased step by step (Sartini et al., 2013). The percentage of multiply innervated muscle cells was considered as an axon sprouting index for each muscle, as previously described (Sartini et al., 2013). At least 50 cells for each muscle were analyzed.

### Cross-sectional area analysis

Muscle fiber CSA was determined in the mid-belly region of soleus muscles, as previously described (Brocca et al., 2010). Briefly, serial transverse sections (10 μm thick) were obtained from soleus muscles of three rats for each experimental group. In this set of experiments, contralateral muscles were considered as undenervated controls. Images of the sections were captured from a light microscope (Leica DMLS) equipped with a camera (Leica DFC 280). Fiber CSA was measured with ImageJ analysis software (NIH, Bethesda, MD, United States) and expressed in square micrometers.

### Western blot analysis

Frozen muscle samples (from *n* = 3 rats for each experimental group as for CSA analysis) were pulverized and immediately re-suspended in a lysis buffer (20 mM tris–HCl, 1% Triton X100, 10% glycerol, 150 mM NaCl, 5 mM EDTA, 100 mM NaF, and 2 mM NaPPi supplemented with 1× protease, phosphatase inhibitors (Sigma-Aldrich) and 1 mM PMSF). The homogenate obtained was kept in ice for 20 min and then centrifuged at 18,000 g for 20 min at 4°C. The supernatant was stored at −80°C until ready to use. Protein concentration was evaluated for each sample, and equal amounts of muscle samples were loaded on gradient precast gels purchased from Bio-Rad (AnyKd; Hercules, CA, United States). After the gel run, proteins were electro-transferred to PVDF membranes at 35 mA overnight. The membranes were incubated in 5% milk for 2 h, rinsed with TBST buffer (0.02 M tris and 0.05 M NaCl, pH 7.4–7.6), and subsequently probed with specific primary antibodies. Thereafter, the membranes were incubated in HRP-conjugated secondary antibody. The primary antibodies used were anti-rabbit p-AMPK^(thr 172)^ and anti-rabbit AMPK (1:1000 Cell Signaling), anti-rabbit PGC-1α (1:1000, Abcam), and anti-rabbit LC3B (1:1000 Sigma-Aldrich). The protein bands were visualized by an enhanced chemiluminescence method in which luminol was excited by peroxidase in the presence of H_2_O_2_ (ECL Select, GE Healthcare). The content of each protein investigated was assessed by determining the brightness–area product of the protein band normalized on the total protein level detected by Red Ponceau staining.

### Statistical analyses

Data were analyzed using GraphPad Prism 9 (GraphPad Software, United States). Each series of data was analyzed with the ROUT method (*Q* = 1%) (Motulsky and Brown, 2006) for detecting outliers that were not included in statistical analysis and graphs. To properly apply parametric or non-parametric statistics, data distributions were explored using the Shapiro–Wilk test, while the homoscedasticity/homogeneity of variance means was checked using Levene’s test. Differences between groups (Run-I *n* = 12, Run-F *n* = 10, Run-S *n* = 10, Sed *n* = 15, Und *n* = 5) were evaluated using one-way or two-way ANOVA analysis followed by Tukey’s *post hoc* for multiple comparisons when appropriate. Significance was accepted at *p* < 0.05. The statistical design for each experimental analyses is detailed in the corresponding figure legend.

## Results

### Electrophysiology

To assess the percentage of multiply innervated muscle fibers, which is usually considered as an axon sprouting index (Sartini et al., 2013), electrophysiological recordings were performed 10 days after soleus denervation.

**FIGURE 2:**
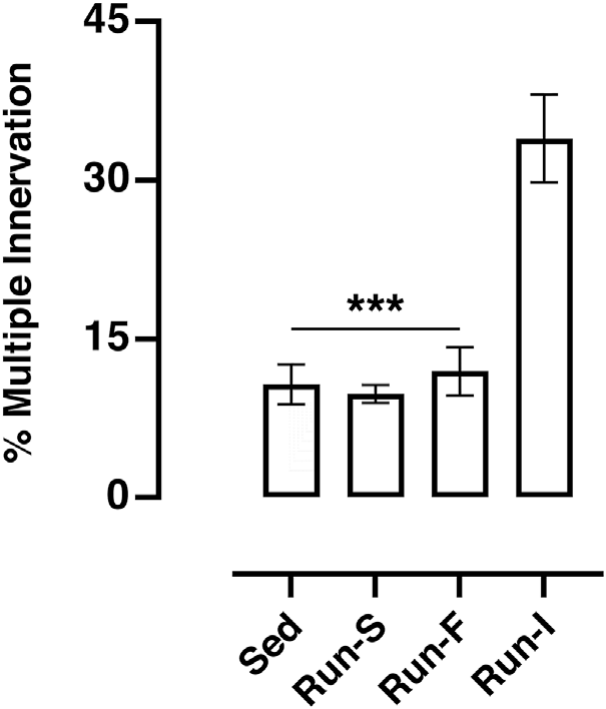
Effect of different exercise protocols on soleus muscle multiple innervation 10 days after nerve crush. One-way ANOVA: *F* (3, 17) = 18.87; Tukey’s *post hoc* test: ***p < 0.001 vs. Run-I. Data are expressed as mean and SEM (Sed *n* = 8, Run-S *n* = 4, Run-F *n* = 4 and Run-I *n* = 5).

Under sedentary condition (Sed), 10.71% (SEM = 1.89) of the muscle fibers was found to be multiply innervated. A similar percentage was also obtained both in Run-S (*M* = 9.80%, SEM 0.84) and in Run-F (*M* = 11.83%, SEM = 2.27) groups. Conversely, the Run-I group showed a significantly higher percentage of multiply innervated muscle fibers reaching, on average, 33.94% (SEM = 4.16; Figure 2).

### *In vivo* tension recordings

To establish whether the different exercise paradigms influence the state of recovery during the reinnervation process, the isometric contractile properties of soleus muscle were evaluated 10 days after nerve crush. To this end, all the nerve-crushed groups were compared to undenervated (Und) rats.

**FIGURE 3:**
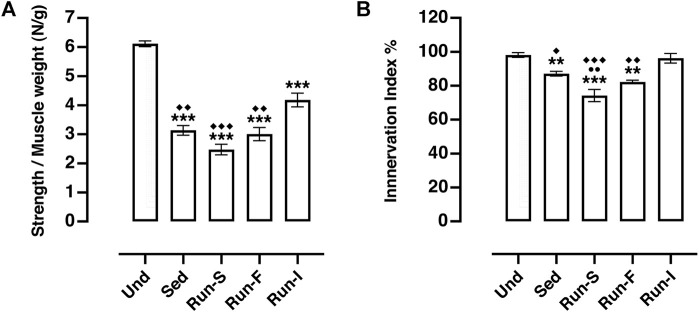
Effect of different exercise protocols on soleus muscle re-innervation 10 days after nerve crush. **(A)** *In vivo* muscle tension recordings obtained by the tetanic stimulation of nerve. One-way ANOVA: *F* (4, 23) = 48.27, *p* < 0.001; Tukey’s *post hoc* test: ***p < 0.001 vs. Und, ^◆◆^ *p* < 0.01 and ^◆◆◆^ *p* < 0.001 vs. Run-I (Und *n* = 5, Sed *n* = 9, Run-S *n* = 4, Run-F *n* = 4 and Run-I *n* = 6). **(B)** Muscle innervation index computed as strength obtained by nerve stimulation/strength obtained by muscle stimulation (both normalized to muscle weight) ratio. One-way ANOVA: *F* (4, 22) = 18.05, *p* < 0.001; Tukey’s *post hoc* test: **p < 0.01 and ***p < 0.001 vs. Und; ^●●^ *p* < 0.01 vs. Sed; ^◆^ *p* < 0.05, ^◆◆^ *p* < 0.01 and ^◆◆◆^ *p* < 0.001 vs. Run-I (Und *n* = 5, Sed n = 8, Run-S *n* = 4, Run-F *n* = 4 and Run-I *n* = 6). Data are expressed as mean and SEM.

Soleus muscle contraction evoked by nerve stimulation in all the experimental groups (Sed: *M* = 3.14 N/g, SEM = 0.17; Run-S: *M* = 2.47 N/g, SEM = 0.18; Run-F: *M* = 3.00 N/g, SEM = 0.22 and Run-I: 4.18 N/g, SEM = 0.24) showed a significant decreased strength compared to Und controls (*M* = 6.12 N/g, SEM = 0.10; Figure 3A). However, rats underwent intermittent exercise protocol (Run-I) expressed a significantly higher muscle strength in response to nerve stimulation than the other nerve-crushed groups (Figure 3A).

The ratio between the muscle strength obtained by nerve stimulation and the strength evoked by direct muscle stimulation was considered as a muscle innervation index (Sartini et al., 2013) (Figure 3B). Analysis of this parameter pointed out that muscle innervation extension appeared to be significantly related to both pattern and intensity of the exercise protocol used. Indeed, among the denervated experimental groups, only the Run-I animals showed muscle innervation index values (*M* = 96.23%, SEM = 2.82) comparable with those collected from the Und controls (*M* **=** 98.14%, SEM = 1.38; Figure 3B). On the other hand, both Run-F (*M* = 82.28%, SEM = 0.99) and Run-S (*M* = 74.21%, SEM = 3.60) groups exhibited an innervation index significantly lower compared to Run-I, but, while the Run-F innervation index was very similar to that gathered in Sed controls (*M* = 87.08%, SEM = 1.37) (Figure 3B), unexpectedly, the Run-S group showed an index of muscle innervation significantly lower even in comparison to Sed controls (Figure 3B).

### Muscle fiber size

**FIGURE 4:**
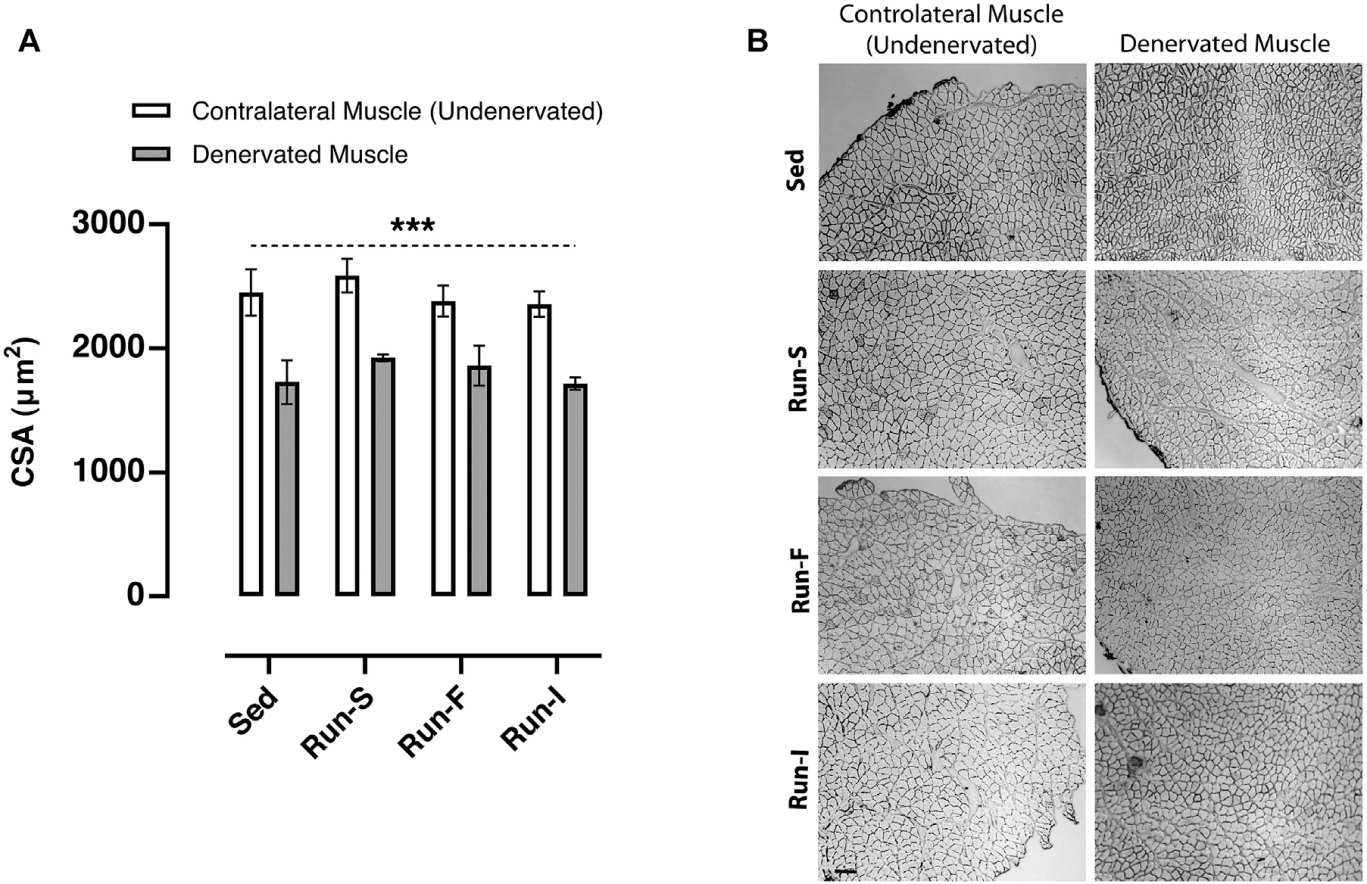
Effect of different exercise protocols on the cross-sectional area of soleus muscle fibers after nerve crush. **(A)** Cross-sectional area determined in Sed, Run-S, Run-F, and Run-I soleus muscle. Two-way ANOVA, main effect type of exercise: *F* (3, 16) = 1.002, *p* = 0.417; main effect of denervation: *F* (1, 16) = 46.57, *p* < 0.001; type of exercise-by-denervation interaction effect: *F* (3, 16) = 0.2077, *p* = 0.890. ***p < 0.001 indicates the main effect of denervation with respect to the contralateral side (undenervated) in each group. Data are expressed as mean and SEM (denervated muscle: Sed *n* = 3, Run-S *n* = 3, Run-F *n* = 3 and Run-I *n* = 3; contralateral muscle: Sed *n* = 3, Run-S *n* = 3, Run-F *n* = 3 and Run-I *n* = 3). **(B)** Representative images of cross-sections of soleus muscles from Sed, Run-S, Run-F, and Run-I experimental groups. Scale bar: 100 *µ*m.

To assess whether the different exercise protocols affect the skeletal muscle fiber size, cross-sectional areas (CSAs) of muscle fibers were determined on soleus cross-sections 10 days after nerve crush. All denervated muscles analyzed from the experimental groups, namely, Sed (*M* = 1726.46 μm^2^, SEM = 306.08), Run-S (*M* = 1923.67 μm^2^, SEM = 48.18), Run-F (*M* = 1859.16 μm^2^, SEM = 279.33), and Run-I (*M* = 1714.11 μm^2^, SEM = 87.33), showed significant smaller CSA values; thus, a significant muscle fiber atrophy compared to the corresponding undenervated contralateral muscle (Sed: *M* = 2451.35 μm^2^, SEM = 321.14; Run-S: *M* = 2585.42 μm^2^, SEM = 236.26; Run-F: *M* = 2380.46 μm^2^, SEM = 213.90 and Run-I: 2375.76 μm^2^, SEM = 180.00; Figures 4A,B).

### AMPK and PGC-1α protein expression

**FIGURE 5:**
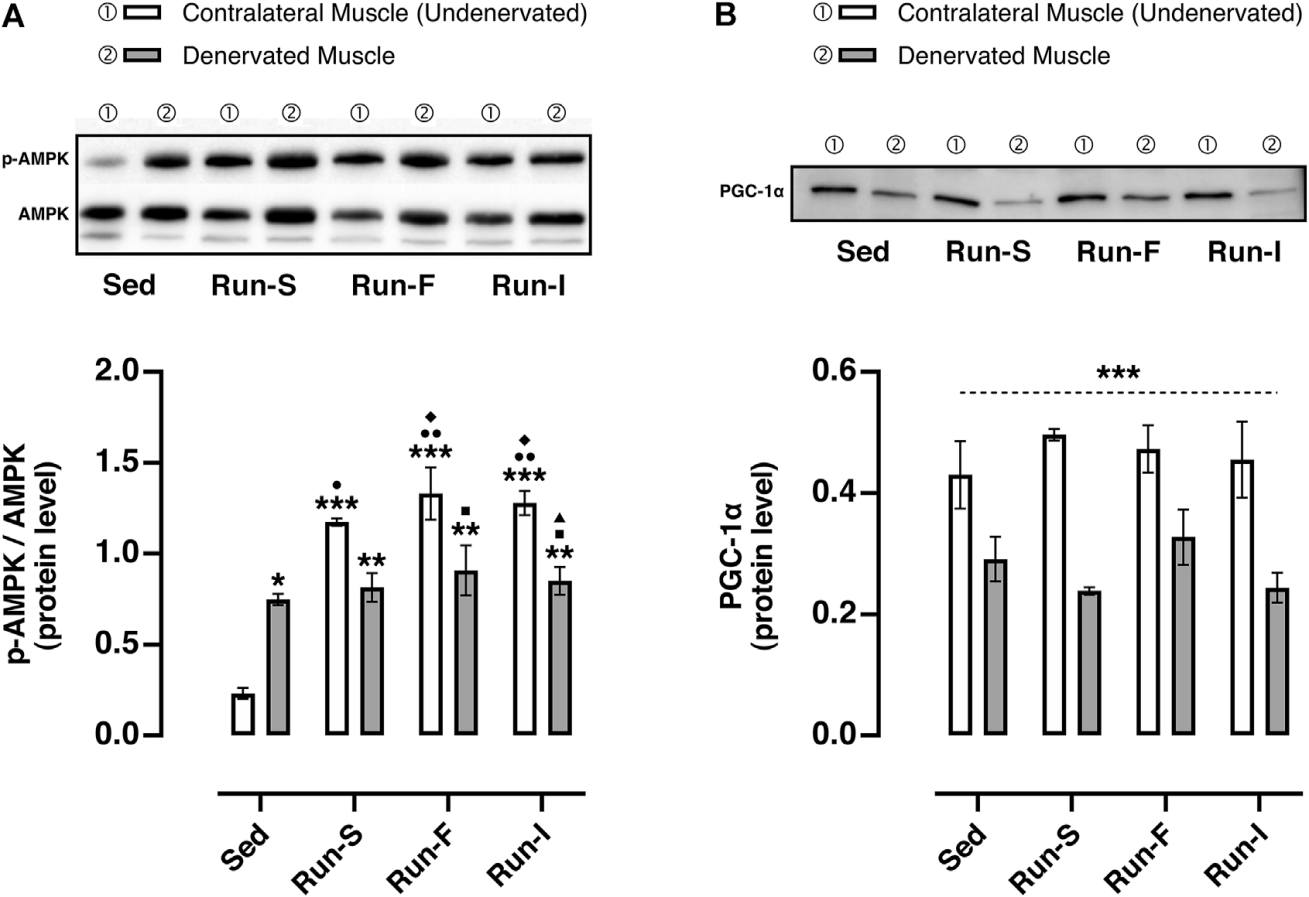
Effect of different exercise protocols on the oxidative metabolism of soleus muscle after nerve crush. **(A)** Representative western blot of AMPK and p-AMPK (top panel); determination of the activation level of AMPK measuring the ratio between the phosphorylated (p) and total forms (bottom panel). Two-way ANOVA, main effect type of exercise: *F* (3, 16) = 22.97, *p* < 0.001; main effect of denervation: *F* (1, 16) = 8.227, *p* = 0.011; type of exercise-by-denervation interaction effect: *F* (3, 16) = 14.55, *p* < 0.001; Tukey’s *post hoc* test: *p < 0.05, **p < 0.01 and ***p < 0.001 vs. Sed (contralateral muscle), ^●^ *p* < 0.05 and ^●●^ *p* < 0.01 vs. Sed (denervated muscle), ^◆^ *p* < 0.05 vs. Run-S (denervated muscle), ^■^ *p* < 0.05 vs. Run-F (contralateral muscle), ^▲^ *p* < 0.05 vs. Run-I (contralateral muscle). **(B)** Representative western blot of PGC-1α (top panel); quantification of protein levels of PGC-1α (bottom panel). Two-way ANOVA, main effect type of exercise: *F* (3, 16) = 0.5976, *p* = 0.626; main effect of denervation: *F* (1, 16) = 44.90, *p* < 0.001; type of exercise-by-denervation interaction effect: *F* (3, 16) = 1.019, *p* = 0.410. ***p < 0.001 indicates the main effect of denervation with respect to the contralateral side (undenervated) in each group. Data are expressed as mean and SEM (denervated muscle: Sed *n* = 3, Run-S *n* = 3, Run-F *n* = 3 and Run-I *n* = 3; contralateral muscle: Sed *n* = 3, Run-S *n* = 3, Run-F *n* = 3 and Run-I *n* = 3).

To estimate the impact of the different exercise protocols on muscle metabolic adaptations, the AMPK activation and PCG-1α protein expression were evaluated in both denervated and contralateral (undenervated) soleus muscles 10 days after nerve crush. In this regard, the activation of AMPK was assessed by the ratio of the active (phosphorylated) and the total form of AMPK (Figure 5A). As expected, the denervated muscles of the Sed group (*M* = 0.80, SEM = 0.03) displayed an increased AMPK activation in comparison with the corresponding contralateral muscles (*M* = 0.23, SEM = 0.03), thus showing a clear denervation effect in triggering AMPK-mediated muscle wasting. Regarding the trained experimental groups, all the denervated muscles (Run-S: *M* = 0.81, SEM = 0.08; Run-F: *M* = 0.90, SEM = 0.13, Run-I: *M* = 0.85, SEM = 0.07) displayed comparable p-AMPK/AMPK values with those collected in the denervated Sed group. Differently, exercise led to a significantly higher activation of AMPK in all the undenervated contralateral muscles of trained rats (Run-S: *M* = 0.81, SEM = 0.08; Run-F: *M* = 0.90, SEM = 0.13, Run-I: *M* = 0.85, SEM = 0.07) in comparison with the corresponding denervated muscles.

Concerning the PCG-1α, all denervated muscles analyzed from the experimental groups, namely, Sed (*M* = 0.29 protein level arbitrary unit, P.A.U., SEM = 0.03), Run-S (*M* = 0.24 P.A.U., SEM = 0.01), Run-F (*M* = 0.33 P.A.U., SEM = 0.04), and Run-I (*M* = 0.24 P.A.U., SEM = 0.02), showed a significant down-regulation of PCG-1α compared to the corresponding undenervated contralateral muscle (Sed: *M* = 0.43 P.A.U., SEM = 0.05; Run-S: *M* = 0.49 P.A.U., SEM = 0.01; Run-F: *M* = 0.47 P.A.U., SEM = 0.04 and Run-I: *M* = 0.45 P.A.U., SEM = 0.06; Figure 5B).

### LC3 autophagy marker

To check whether the different exercise paradigms influence the activity of the autophagy system, which mediates many of the beneficial effects of exercise (Lira et al., 2013) and affects muscle–nerve interaction (Carnio et al., 2014), the LC3-II/LC3-I ratio, commonly used as a marker for the induction of autophagy, was determined. Among all the experimental groups, the Run-I showed a significant increase of the LC3-II/LC3-I values in both denervated (*M* = 5.07, SEM = 0.14) and undenervated contralateral muscles (*M* = 2.04, SEM = 0.01) compared to all the other experimental conditions (Sed denervated: *M* = 1.20, SEM = 0.22; Sed contralateral: *M* = 0.72, SEM = 0.21; Run-S denervated: *M* = 0.99, SEM = 0.09; Run-S contralateral: *M* = 0.73, SEM = 0.04; Run-F denervated: *M* = 1.16, SEM = 0.01; Run-F contralateral: *M* = 0.61, SEM = 0.03).

## Discussion

The present study aimed to gain insight into the impact of exercise on muscle reinnervation and its functional recovery following peripheral nerve injury by using a nerve-crushed animal model. Since the effects of exercise can be influenced by several parameters, such as the amount and intensity of training, we tested different protocols of exercise at the beginning of the muscle reinnervation process, upon peripheral nerve crush and nerve regeneration, and we adopted a multidisciplinary approach for evaluating functional and metabolic outcomes. The key findings in our study are as follows: 1) different exercise protocols differentially affect the reinnervation process following denervation; 2) exercise delivered in intermittent mid-intensity treadmill training enhances soleus muscle reinnervation, whereas a slow continuous running protocol worsens the functional outcome; 3) these functional effects are not mirrored by the corresponding metabolic effects on the soleus muscle; 4) the activity of the autophagy system is boosted by the intermittent running protocol only.

Traumatic peripheral nerve injuries represent a critical and demanding public health issue that affects an increasing number of individuals each year with a marked impact on the quality of life (Rodrigues et al., 2012; English et al., 2014; Bergmeister et al., 2020). In this regard, it is well documented that damaged peripheral nerves may regenerate after traumatic injuries, but usually, the regenerative process is slow and full functional recovery is rarely reached (English et al., 2014). Actually, despite the continuing progress in understanding the pathophysiology of the peripheral nervous system and advancements in surgical techniques of peripheral nerve repair, full functional recovery is still the most challenging problem related to peripheral nerve injury treatment and rehabilitation (Maugeri et al., 2021). In this context, using non-surgical approaches complementary to surgery to boost recovery and the reinnervation process is of primary interest (Martínez de Albornoz et al., 2011; Maugeri et al., 2021). Among non-surgical therapies for traumatic peripheral nerve injuries, compelling evidence has highlighted the effectiveness of the “activity-related therapies,” such as electrical stimulation and exercise, in boosting axonal regeneration and reinnervation of the target muscle (Geremia et al., 2007; Sabatier et al., 2008; Asensio-Pinilla et al., 2009; Sartini et al., 2013). Notably, among the activity-related therapies, there has been a growing interest in evaluating the effects of exercise on peripheral nerve injuries in light that it is relatively riskless and provides pleiotropic effect in promoting metabolic and psychological well-being by ameliorating general health besides boosting the functional recovery upon traumatic injuries (Maugeri et al., 2021). However, exercise is not a *panacea* per se. Indeed, the adopted type of exercise dramatically determines the outcomes of the experimental rehabilitation therapy (Maugeri et al., 2021). Thus, finding the proper activity protocol at the service of functional recovery is fundamental.

In this scenario, our electrophysiological and *in vivo* tension recording data collectively pose the pattern and the intensity of exercise as crucial impacting factors on functional muscle recovery upon denervation. Indeed, here we demonstrate the effectiveness of intermittent mid-intensity exercise as a promising paradigm to improve muscle recovery following a peripheral nerve injury. Notably, in comparison with the other applied exercise protocols (Run-S and Run-F), mid-intensity intermittent running (Run-I) has emerged to be the more suitable pattern of activity for boosting axonal sprouting during the reinnervation process (Figures 2, 3). These findings confirm and extend our previous work (Sartini et al., 2013) and are in line with Willand et al. (2015), where an intermittent electrical stimulation of muscle effectively enhanced functional recovery following nerve transection and repair in rats. Conversely, exercise protocols consisting of continuous running patterns fail to stimulate the multiple innervation enhancement of muscle fiber prodromal to improve muscle reinnervation at the service of functional recovery upon the injury (Figures 2, 3). In particular, we found that a slow-intensity continuous treadmill-training (Run-S) worsens the functional outcome, inducing a further reduction in the soleus muscle strength evoked by tetanic stimulation of nerve even in comparison to nerve-crushed rats maintained in sedentary conditions (Sed; Figure 3).

Collectively, these findings would suggest the existence of an exercise intensity threshold below which muscle-nerve interplay becomes not only inefficient but even detrimental for functional recovery, posing the intermittence of the training protocol as a necessary condition to induce the beneficial effects prodromal to complete muscle reinnervation, thus a more efficient functional recovery. In this view, it is worth mentioning that, as occur in muscle innervation development, several young newly formed synapses are silent during the muscle reinnervation process, and differential electrical activity may affect their maturation and functioning (Dennis and Miledi, 1974; Colman et al., 1997; Personius and Balice-Gordon, 2001; Buffelli et al., 2002; Tomas et al., 2011). Along this line of reasoning, we may hypothesize that a low-frequency nerve stimulation occurring during slow-intensity continuous running does not allow silent synapses to become active, inducing the loss of newly formed terminals and thus the synapse pruning. Contrarily, a mid-intensity intermittent pattern of neuromuscular activity, like the one adopted in Run-I nerve-crushed rats, may promote a specific firing pattern capable of strengthening, thus stabilizing these newly formed terminals (Personius and Balice-Gordon, 2001; Buffelli et al., 2002).

Although a clear beneficial role of the mid-intensity intermittent running paradigm on the reinnervation process has emerged by functional analysis, this functional effect was not mirrored by enhancing the key metabolic mediator of exercise-induced metabolic adaptations. In this regard, as shown in our previous work (Sartini et al., 2013), the exercise-induced axonal sprouting, prodromal to complete reinnervation of the denervated muscle, reaches its acme 10 days upon denervation with 6 days of training. However, if on one side this specific time interval has proven to be suitable concerning the reinnervation process, which is the primary focus of the current work, on the other side, it could be too short to counteract the denervation-induced metabolic alterations and muscle atrophy (Figure 5).

In this regard, looking at the effects on skeletal muscle, denervated muscles exhibited a decrease of fiber size, high activation levels of AMPK, a key sensor of energy status (Herzig and Shaw, 2018), and low PGC-1α protein content, a key regulator of energy metabolism (Fernandez-Marcos and Auwerx, 2011), thus showing complete agreement with alterations reported in response to nerve crush (Muller et al., 2007; Yue et al., 2019).

AMPK and PGC-1α are critical for the beneficial cellular adaptations of endurance exercise (Jung and Kim, 2014). Accordingly, although all exercise modalities effectively increased AMPK activation in non-denervated muscle, no changes in PGC-1α content were observed. This apparent discrepancy could be related to the time interval selected for the experimental analysis. Similarly, all the exercise paradigms failed to counteract nerve injury-induced PGC-1α deficit and consequently muscle atrophy, in accordance with the notion that high levels of PGC-1α are necessary to protect muscle from denervation atrophy (Sandri et al., 2006).

Exercise training-induced skeletal muscle adaptation requires both the addition and clearance of cellular components. In this context, autophagy seems to be involved in mediating many of the beneficial effects of exercise (Halling and Pilegaard, 2017) by ensuring normal physiological activities of skeletal muscle with exercise training (Lira et al., 2013). Furthermore, autophagy inhibition leads to destabilization of muscle–nerve interaction while its recovery improves neuromuscular junction morphology (Carnio et al., 2014).

**FIGURE 6:**
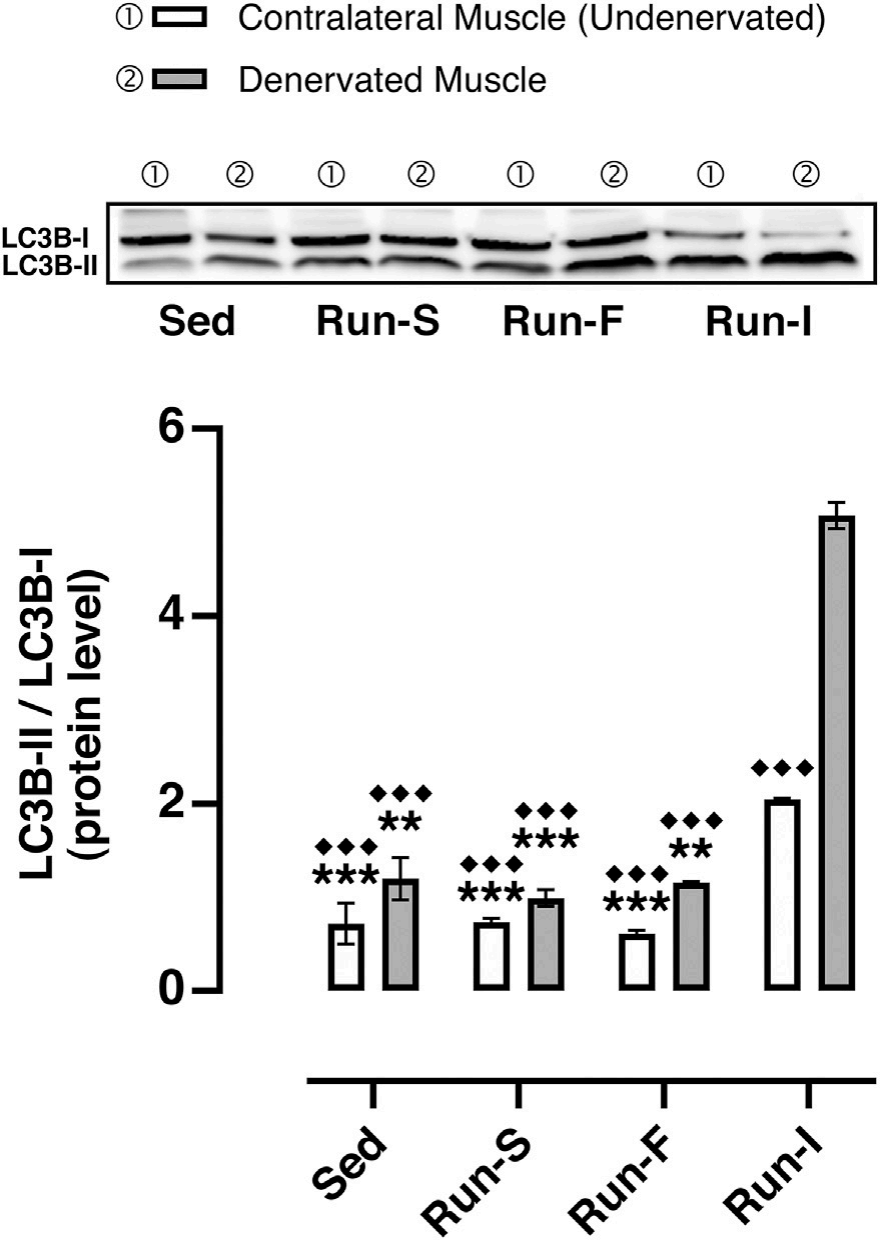
Effect of different exercise protocols on soleus muscle autophagy machinery after nerve crush. Representative western blot of LC3B-I and LC3B-II (top); quantification of protein levels of LC3B-II based on the ratio between the content in forms II and I of LC3B (bottom). Two-way ANOVA, main effect type of exercise: *F* (3, 16) = 218.3, *p* < 0.001; main effect of denervation: *F* (1, 16) = 143.4, *p* = 0.011; type of exercise-by-denervation interaction effect: *F* (3, 16) = 52.55, *p* < 0.001; Tukey’s *post hoc* test: **p < 0.01 and ***p < 0.001 vs. Run-I (contralateral muscle), ^◆◆◆^ *p* < 0.001 vs. Run-I (denervated muscle). Data are expressed as mean and SEM (Denervated Muscle: Sed *n* = 3, Run-S *n* = 3, Run-F *n* = 3 and Run-I *n* = 3; Contralateral Muscle: Sed *n* = 3, Run-S *n* = 3, Run-F *n* = 3 and Run-I *n* = 3).

Our results show that the increase of autophagy responds specifically to the mid-intensity intermittent running exercise. This finding is in line with the concept that the mechanism behind autophagy induction may depend on factors such as intensity/duration of exercise (Schwalm et al., 2015). It is noteworthy that this exercise pattern increases autophagy-related marker LC3 not only in denervated but also in contralateral non-denervated soleus muscles, suggesting a targeted effect of this exercise pattern independent of muscle conditions (Figure 6). Of note, the activation of autophagy during the mid-intensity intermittent running exercise would seem to occur with a mechanism independent of the AMPK pathway (Herzig and Shaw, 2018), as AMPK activation is not regulated differently in the three different exercise programs. Furthermore, it is also known that an excess of autophagy can be detrimental, leading to the loss of muscle mass (Sandri, 2010). We exclude that in our condition, the increase of autophagy may have a role in this process since fiber CSA was found to be unchanged.

Considering the positive correlation between the autophagy marker and the functional improvement observed with the mid-intensity intermittent running exercise, it is reasonable to speculate that the induction of autophagy may have contributed to the functional improvement promoting muscle–nerve interaction stability.

## Conclusion

Complete functional recovery following peripheral nerve injuries is still the most challenging problem related to treatment and rehabilitation. In our study, we assessed different exercise protocols at the beginning of the muscle reinnervation process, upon peripheral nerve crush and nerve regeneration and investigated their effects on reinnervation. A mid-intensity intermittent running exercise enhances functional recovery by boosting axonal sprouting and muscle autophagy induction, thus promoting muscle–nerve interaction stability. However, it is noteworthy that various factors challenge the effective translation of promising results gathered in pre-clinical animal experimentations. In this regard, the post-injury interval adopted in the present work, namely, 10 days, could not reflect the temporal full extent of the natural recovery of patients. Nonetheless, the present findings are encouraging, and, in future studies, this exercise protocol could be exploited to ameliorate recovery in humans following peripheral nerve injuries.

## Data Availability

The original contributions presented in the study are included in the article; further inquiries can be directed to the corresponding author.
